# Understanding the influence of socioeconomic status on children physical disciplinary practices in Bangladeshi households

**DOI:** 10.1371/journal.pone.0320239

**Published:** 2025-04-10

**Authors:** Mahnaz Ibrahim, Md Hridoy Patwary

**Affiliations:** 1 Institute of Statistical Research and Training, University of Dhaka, Dhaka, Bangladesh; Vytautas Magnus University, LITHUANIA

## Abstract

**Background** Despite progress in reducing violence against children, physical disciplinary practices remain prevalent in many parts of the world, including Bangladesh. Understanding the sociodemographic factors contributing to these practices is crucial for developing effective interventions to protect children from violence. This study aims to estimate the prevalence and impact of household economic status on the physical disciplinary practices experienced by children under five years old in various sociodemographic contexts.

**Methods** Data from the Bangladesh Multiple Indicator Cluster Survey (MICS) 2019 was used to explore physical disciplinary practices against children under five, focusing on factors such as household wealth, region, type of residence, child’s gender, and mother’s education level. Logistic regression analysis was employed to examine the adjusted association between sociodemographic factors and physical disciplinary practices.

**Findings** The study found that 64.88% of children under five experienced physical disciplinary practices at home. The odds of being subjected to PDP were higher for children from lower wealth index categories (poorest, poorer, middle, richer) compared to those in the richest category, with AORs ranging from 1.21 to 1.35 and all p-values <0.01. Urban residence was associated with increased odds of being physically punished (AOR: 1.09, 95% CI: 1.01–1.20). Boys had higher odds of being physically punished compared to girls (AOR: 1.15, 95% CI: 1.07–1.23). The odds of being physically punished were greater for children of less-educated mothers. Furthermore, mothers who believed that physical punishment was necessary for child-rearing had higher odds of using such practices (AOR: 2.10, 95% CI: 1.94–2.27).

**Conclusion** Socioeconomic factors, especially household wealth, significantly influence the risk of experiencing physical disciplinary practices among children. The findings emphasize the need for targeted interventions to protect vulnerable children, particularly in urban and impoverished settings.

## Introduction

Child discipline aims to teach reasonable and appropriate behaviors while addressing negative behavioral patterns, fostering self-control, and guiding them to become responsible and socially adept adults [1,2]. The three primary strategies for handling child misbehaviour are discussion and reasoning, verbal or non-verbal aggression, and violence [3]. Violence against children can be all forms of emotional, sexual, and physical violence, neglect, negligent treatment, and exploitation and can occur anywhere including home, schools, playgrounds, day-cares, etc. In Bangladesh, domestic violence against children is one of the most widespread types of child maltreatment [4]. This study focuses on the physical violence against children at home that may come as disciplinary actions perpetrated by parents or other caregivers in Bangladesh.

Numerous studies have highlighted the inefficiencies of violent practices and their detrimental effects on children, including developmental problems and damaged child-parent relationships [5–10]. These practices also contribute to increased hostility [11,12], low self-esteem [5,12], major depressive episodes [10,12], conduct disorder, attention deficit hyperactivity disorder, socialized aggression [10–12], and even death [13–15]. Childhood maltreatment also increases the risk of mental health issues in later life and perpetuates intergenerational cycles of abuse by influencing parenting practices toward neglectful or abusive behaviors [16,17]

Violent disciplinary practices have been prohibited in many countries as a result [18]. Besides, these practices can be viewed as a form of a child’s rights violation. Globally, it is estimated that approximately 1 billion children aged 2–17 years experienced violence or neglect in the year preceding the surveys [19,20]. Child maltreatment is a widespread global issue that impacts the lives of millions of children worldwide [21]. In contrast, nonviolent approaches have many benefits for child development. For example, the MISC approach in Ethiopia improved maternal sensitivity and children’s cognitive and socioemotional outcomes by promoting nurturing, non-violent interactions [22]. Similarly, the Learning Through Play program in Pakistan enhanced mothers’ knowledge and attitudes about infant development through play-based activities, fostering positive parent-child interactions [23].

In many countries, efforts to reduce corporal punishment have proven effective. For example, Nepal, the first South Asian country to ban all corporal punishment in 2018, implemented public awareness campaigns and parenting education to promote non-violent discipline [24]. Similarly, Sri Lanka has integrated non-violent discipline training into teacher education programs to curb corporal punishment in schools [25]. Outside the region, countries like Uruguay and South Africa have strengthened legal frameworks, accompanied by educational campaigns, to promote positive disciplinary practices and reduce physical violence against children [26,27].

Studies conducted across various countries have examined violent disciplinary practices from multiple perspectives. While some research has focused on developing reliable tools to measure these practices [28–31], others have highlighted the prevalence of violent disciplinary measures. Numerous studies reveal that such practices persist in many countries, including Bangladesh [32–37]. However, this issue often goes unnoticed in Bangladesh due to a lack of awareness, knowledge, and the persistence of harmful practices.

Bangladesh is a developing nation with rapid economic growth, yet poverty remains a significant challenge [38]. Almost 20% of the population lives below the national poverty line, and income disparities create challenges in accessing basic needs such as education, healthcare, and nutrition [39,40]. Consequently, social malpractices often go unnoticed amidst the broader struggle for survival [41]. Many parents do not perceive violent discipline as an injustice or a violation of children’s rights. Instead, they view it as a normal and acceptable method for teaching proper behavior. This can be attributed to various factors, including the social acceptance and tacit condoning of certain forms of violence against children, which are not always perceived as abusive. These perceptions are deeply ingrained, perpetuated across generations, and reinforced by a lack of awareness about the long-term negative consequences of such practices. Many victims of these violences are too young or vulnerable to share their experiences, and some may feel compelled to hide incidents of violence and abuse, particularly when the perpetrators are individuals they know and trust. These societal norms and perceptions are often intertwined with a household’s socioeconomic status. Families facing economic hardships may experience heightened stress and limited access to resources, which can influence parenting practices, including the use of physical punishment. Even though there exist many studies on poverty and socioeconomic status and how it impacts the quality of life, there is still a gap in the literature regarding the relationship between children’s physical punishment at home and the socioeconomic status of households in Bangladesh.

In Bangladesh, a study examined the relationship between socioeconomic characteristics and child corporal punishment revealing that approximately 87% of children experience punishment at school, with the most common form being physical punishment [42]. The study also found a strong correlation between socioeconomic status and the likelihood of corporal punishment [42]. Among the punished children, 92.3% come from poor families, where parents typically work as farmers or day laborers, compared to only 7.7% from non-poor families [42]. Fortunately, in 2010, the Education Ministry issued a directive prohibiting all forms of corporal punishment for students [43]. In 2011, the ministry issued an additional circular outlining a policy aimed at eliminating corporal punishment [43]. So, corporal punishment in schools or educational institutions is decreasing day by day and hopefully, it will be unrooted. Also, Bangladesh has made significant strides in establishing legal frameworks to safeguard children’s rights. The Children Act 2013 serves as a comprehensive legal instrument aimed at protecting children from violence, abuse, exploitation, and neglect. The act emphasizes the prohibition of physical, emotional, and sexual violence against children and establishes mechanisms such as child welfare boards and specialized child courts [44]. In 2001, the United Nations initiated the first comprehensive global study on all forms of violence against children [45]. The study thoroughly analyzed a range of violent behaviors, encompassing physical violence, neglect, sexual violence, harmful traditional practices, and psychological violence. A key finding was that most violence against children occurs at home[46]. Still, physical punishment at home by caregivers goes untackled and overlooked in Bangladesh. Even though most of the deaths of the child were a result of the severe physical violence at home [13,14].

Despite extensive research on child abuse and corporal punishment, limited studies have explicitly examined the link between socioeconomic status and physical disciplinary practices against children within Bangladeshi households at national level. This study specifically aims to fill this research gap by analyzing the influence of socioeconomic status on the prevalence of physical disciplinary practices within Bangladeshi households. The vulnerable cohort, that is, the households where physical punishment is mostly practiced and consequently violence against children is occurring is identified based on the socioeconomic and sociodemographic attributes using the Bangladesh Multiple Indicator Cluster Survey (MICS 2019). This should help the Sustainable Development Goals, specifically goal 16.2 of the 2030 Agenda to “end abuse, exploitation, trafficking and all forms of violence against, and torture of, children” by assisting in designing intervention strategies [47]. Particularly, the study would give (a) estimates of overall physical disciplinary practices against under-5-year-old children in different sociodemographic settings and (b) an estimate of the impact of the economic status of a household on physical disciplinary practices.

## Materials and methods

### Data overview

The study drew on data from the Bangladesh MICS, a household survey program conducted in 2019 by the Bangladesh Bureau of Statistics (BBS) in partnership with UNICEF Bangladesh and can be accessed through https://mics.unicef.org/surveys. Technical support was provided by UNICEF, while UNFPA Bangladesh contributed financial resources for quality assurance visits during data collection which ensures data comparability across countries and time. MICS surveys provide crucial data to guide policies, programs, and national development plans, while also tracking progress towards the Sustainable Development Goals (SDGs) and other global commitments [47].

The 2019 Bangladesh MICS survey was designed to provide estimates on a wide range of indicators related to the well-being of children and women. The survey aimed to generate representative data at the national level as well as for urban and rural areas, the eight administrative divisions, and all sixty-four districts of Bangladesh. It employed a two-stage stratified sampling design to ensure comprehensive coverage. In the first stage, urban and rural areas within each district served as the primary sampling strata. A specified number of census enumeration areas (clusters) were then selected systematically from each stratum using probability proportional to size, based on household counts from the 2011 Population and Housing Census. This approach ensured that larger clusters had a proportionally higher chance of being included in the sample. In the second stage, a household listing was conducted within each selected enumeration area, and 20 households per cluster were systematically chosen. This resulted in a final sample of 3,220 primary sampling units (PSUs) and 64,400 households across the country. By using this stratified sampling design, the survey achieved a wide geographic representation and improved the reliability of estimates for national, urban/rural, and regional levels Because the sample was not self-weighted, appropriate sample weights were applied during analysis to ensure representativeness and accurate estimates at all stratification levels.

For the current study, the authors accessed the 2019 Bangladesh MICS data in July 2024, with a specific focus on the Child Discipline Module. This module provided detailed information about disciplinary practices, allowing for the analysis of the association between socioeconomic status and physical disciplinary methods within Bangladeshi households. In households with more than one child under five, a random selection process was employed. This process used a tool that matched the number of children in the household to the last digit of the survey number assigned to the household questionnaire, ensuring a random yet systematic selection. The name and line number of the selected child were recorded to link disciplinary practice data with other household and child-specific survey information. The interviewer then asked the mother or primary caregiver a series of standardized questions about the disciplinary methods used by household members in the month preceding the interview.

### Ethical approval

The study did not require ethical review approval because it utilized de-identified, publicly available MICS 2019 data. However, the technical committee of the Government of Bangladesh which is led by BBS, approved the survey protocol. A protection protocol was included in the survey protocol to outline the potential risks during the life cycle of the survey and management strategies to abate the risks. Moreover, verbal consent was collected for each of the participating respondents. The respondents were notified by the interviewers that participation in the survey was completely voluntary and all the provided information would be kept confidential and anonymous. Respondents had the right to repudiate answering all or particular questions, as well as to end the interview at any given time.

### Variables

#### Dependent variable.

Physical disciplinary practice at home is the outcome variable in the current study. The response variable was formulated using data from the 2019 Bangladesh MICS, particularly from the Child Discipline Module, which provided comprehensive information on disciplinary practices. To group the mother’s or other primary caregiver’s act of physical violence as the Physical disciplinary practice, the MICS 2019 report along with the MICS standard recode manual were followed. The binary outcome variable Physical disciplinary practice was created by taking the mother’s or primary caregiver’s answers to the following questions into account: (a) spanked, hit, or slapped (him/her) on the bottom with a bare hand; (b) Hit (him/her) on the bottom or elsewhere on the body with something like a belt, hairbrush, stick or other hard object; (c) Hit or slapped (him/her) on the face, head or ears.; (d) Hit or slapped (him/her) on the hand, arm, or leg; (e) Beat (him/her) up, that is hit (him/her) over and over as hard as one could. A positive answer to any one of these questions was taken as ‘yes’ for Physical disciplinary practice at home and the rest were classified as ‘no’. This approach captures the full spectrum of physical disciplinary behaviors, from less severe actions like spanking to more severe forms such as repeated beating, reflecting their shared intent to correct or control a child. It also aligns with the broader understanding of violence, recognizing that all such acts, regardless of severity, can have significant implications for a child’s well-being

#### Independent variables.

Socioeconomic status or wealth index (poorest, poorer, middle, richer, richest) is the main independent variable. It was pre-calculated in the data using principal component analysis on the household assets. The sociodemographic confounding variables which are commonly identified as significant predictors of child discipline practices were selected for this study. Sociodemographic factors that were selected are types of residence (urban, rural), division (Barisal, Chittagong, Dhaka, Khulna, Rajshahi, Rangpur, Sylhet), gender of the child (male/female), mother’s education level (no education, primary, secondary, higher), Parental attitude towards physical disciplinary practices (necessary, not necessary). All the independent variables were derived directly from the 2019 Bangladesh MICS survey data.

### Statistical analyses

The data were weighted using individual weights to address unequal selection probabilities and adjusted for strata and cluster variations to account for the survey’s complex sampling design, ensuring representativeness and accurate standard error estimation. Afterward, univariate analysis was employed to assess the distribution of the sociodemographic variables used in the analysis. The percentage distribution of physical disciplinary practices at home over different factors was then calculated and tested using chi-square to assess any association between the study factors and the outcome which helps to identify statistically significant factors that could be included in the final model. A spatial distribution of physical disciplinary practices among children was also analyzed to assess the district-level pattern. The primary outcome of interest, physical disciplinary practices, was modeled using a generalized linear model (GLM) with a logit link function as it is well-suited for binary outcomes and allows adjustment for confounding variables to estimate odds ratios accurately. The calculated adjusted effects (AORs) along with their statistical significance and 95% confidence intervals were provided. Throughout the study, a statistical significance level of 5% was considered. Missing data were handled using a complete case analysis approach, assuming a missing completely at random (MCAR) mechanism, which involved analyzing only cases with complete data for all relevant variables. All analyses were conducted using Stata V.14.0 (Stata SE V.14, Stata Corp). The spatial map was constructed using R (version 4.1.1). The district-level Shapefile of Bangladesh was accessed from the *bangladesh* [48] package in R, and the map was plotted using *ggplot2*.

## Results

### Descriptive analysis

The prevalence of physical disciplinary practices was 64.88% (n = 9452) suggesting that in most of the households in Bangladesh, PDP is alarmingly common. For instance, a systematic review indicated that in Southeast Asia, the prevalence of corporal punishment among school-aged children ranges from 50% to 60% [49]. In Tanzania, a study found that approximately 51% of primary caregivers reported using harsh physical discipline, which aligns closely with the aforementioned prevalence [50]. This suggests that the rates of physical discipline in these regions are comparably high, indicating a potential cultural normalization of such practices. Furthermore, findings from a UNICEF survey indicated that half of the children in a 33-country study reported having been physically punished by their parents, with the prevalence in the U.S. being even higher, where two-thirds of young children experienced spanking [51]. This comparison emphasizes that while the 64.88% prevalence of physical disciplinary violence is notably high, it is part of a larger global pattern where physical discipline remains a common practice, particularly in certain cultural contexts.

**Table 1:** Percentage distribution of physical disciplinary practices against children by explanatory variables.

Variables	Categories	N (%)	PDP (%)	p-value
**Wealth index**	Poorest	5755 (24.91)	67.97	<0.001
	Poorer	4383 (20.94)	67.44	
	Middle	4352 (18.84)	67.65	
	Richer	4310 (18.66)	65.66	
	Richest	3844 (16.64)	65.94	
**Division**	Barisal	2260 (9.15)	52.22	<0.001
	Chittagong	5129 (20.78)	65.39	
	Dhaka	4888 (19.80)	66.75	
	Khulna	3441 (13.94)	73.69	
	Mymensingh	1448 (5.87)	66.00	
	Rajshahi	2568 (10.40)	66.78	
	Rangpur	2876 (11.65)	62.37	
	Sylhet	2076 (8.41)	68.48	
**Sex of child**	Male	12702 (51.45)	67.90	0.001
	Female	11984 (48.55)	63.84	
**Types of residence**	Urban	4603 (18.65)	64.70	0.150
	Rural	20083 (81.35)	66.28	
**Mother’s education**	No education	2701 (10.94)	66.90	<0.001
	Primary	5842 (23.67)	68.90	
	Secondary	12184 (49.36)	66.69	
	Higher	3958 (16.03)	58.26	
**Parental attitude towards physical disciplinary practices**	Yes, it is necessary	5038 (34.58)	74.39	<0.001
	No, not necessary	9531 (65.42)	58.52	

**Note:** PDP = Physical Disciplinary Practices

Table 1 demonstrates a clear relationship between physical disciplinary practices and the socioeconomic status of a child’s family in Bangladesh. The data revealed that 67.97% of children from the poorest households experience physical disciplinary practices, compared to 65.94% of children from the wealthiest households.

**Fig 1:**
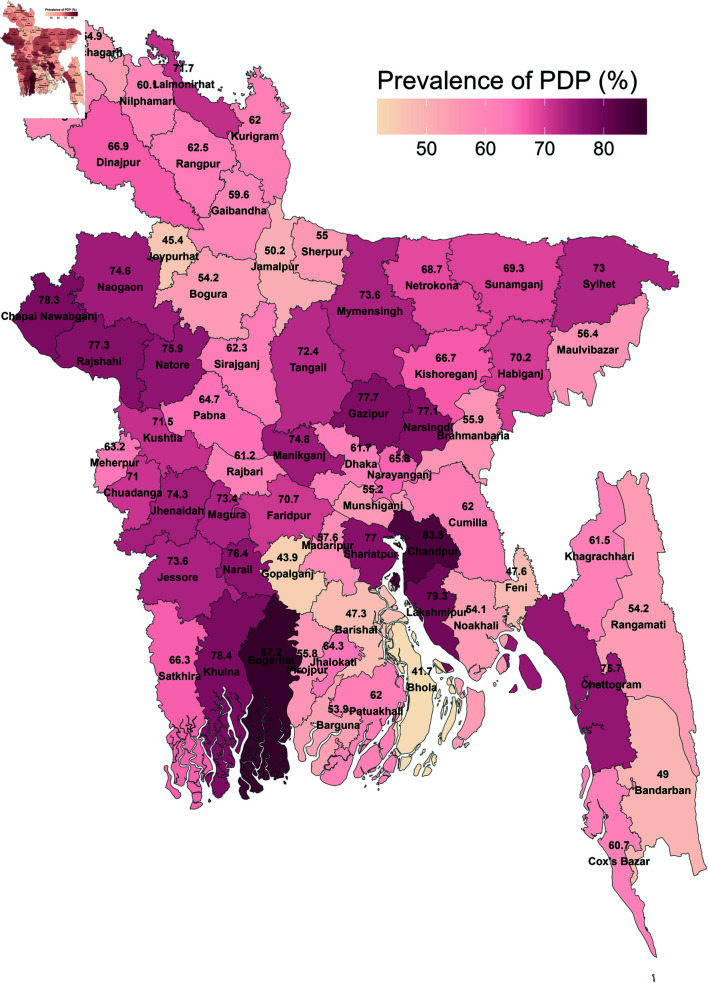
Spatial distribution of physical disciplinary practices (PDP) against children across districts in Bangladesh.

The spatial map in Fig 1 illustrates the spatial distribution of physical disciplinary practices (PDP) against children across districts in Bangladesh. Notably, the northern regions exhibit a lower prevalence, whereas the southern regions show a higher prevalence of PDP. The distribution of respondents across different division was dispersed. Most of the respondents were from Chittagong while the least were from Barisal. Regionally, the prevalence of physical disciplinary practices against children was highest in Khulna division (73.69%), followed by Sylhet division (68.48%). The rates are similar across Dhaka, Mymensingh, and Rajshahi divisions, ranging from 66% to 67%. Chittagong exhibits a slightly lower prevalence at 65.39%. Notably, Rangpur shows a significant reduction in the risk at 62.37%, with Barisal division presenting the lowest risk at 52.22%.

Even though the distribution of the children’s gender in the study was equal, the analysis highlighted a gender disparity in experiencing physical disciplinary practices, with male children (67.9%) being more frequently punished than female children (63.84%).

Moreover, there is a strong association between physical disciplinary practices and maternal education. Children from well-educated families (58.26%) are less likely to experience PDP compared to families with no formal education attained (66.9%). This suggests that higher maternal education is associated with a decrease in the use of physical punishment.

Finally, the data suggests a significant association between parental attitudes towards PDP and its application. Mothers or caregivers who believed that PDP is essential for proper child rearing are more likely to administer such punishment, in contrast to those who do not share this belief.

### The generalized linear model

Socioeconomic status of the household, division the family resides in, sex of the child, mother’s education level, and attitude towards physical disciplinary practices were found to be significantly associated with the likelihood of physical disciplinary practices in a household in the GLM Table 2. Notably, among these factors, parental attitudes towards PDP emerged as the strongest predictor, significantly influencing the prevalence of such practices, followed by geographical division, socioeconomic status, and maternal education level.

Children from the poorest families (AOR 1.34 with 95% CI: [1.17, 1.53]) were more prone to facing physical disciplinary practices than the richest families. Similarly, the likelihood of physical disciplinary practices also significantly increased for children who belonged to the poorer (AOR 1.28), middle (AOR 1.35), and richer (AOR 1.21) wealth group households compared to children from the richest households. Children residing in Khulna and Sylhet had almost 3 times significantly higher odds of experiencing PDP compared to those in Barisal. In Dhaka, Chittagong, Rajshahi, Mymensingh, and Rangpur the odds were significantly higher, almost 2 times higher, than in Barisal. Male children were 15% more likely than female children to face PDP (p-value <0.001). In households that were in urban areas, the odds of PDP against children increased 1.09 times compared to households in rural areas. The children with mothers attaining no (AOR 1.07) or lower level of education, such as primary

(AOR 1.30) and secondary (AOR 1.25), were at higher risk of experiencing PDP compared to those who had mothers with a higher level of formal education. Mothers or caregivers who held a positive attitude towards physical disciplinary practices increased the odds 2.1 times for the children facing physical violence compared to those who do not share this unfounded belief.

**Table 2:** Estimated effects and 95% CI of binary logistic regression model for PDP.

Variables	Categories	Adjusted OR	p-value	95% CI
**Wealth index**	Poorest	1.34	<.001	[1.17, 1.53]
	Poorer	1.28	<.001	[1.12, 1.46]
	Middle	1.35	<.001	[1.19, 1.53]
	Richer	1.21	<.001	[1.08, 1.36]
	Richest*			
**Division**	Chittagong	2.00	<.001	[1.71, 2.35]
	Dhaka	2.05	<.001	[1.75, 2.41]
	Khulna	3.05	<.001	[2.56, 3.64]
	Mymensingh	1.87	<.001	[1.55, 2.25]
	Rajshahi	2.17	<.001	[1.83, 2.57]
	Rangpur	1.68	<.001	[1.42, 2.00]
	Sylhet	2.99	<.001	[2.47, 3.62]
	Barisal*			
**Sex of child**	Male	1.15	<.001	[1.07, 1.23]
	Female*			
**Types of residence**	Urban	1.09	0.050	[1.01, 1.20]
	Rural*			
**Mother’s education**	No education	1.07	0.329	[0.93, 1.24]
	Primary	1.30	<.001	[1.15, 1.47]
	Secondary	1.25	<.001	[1.13, 1.39]
	Higher Secondary or higher*			
**Parental attitude towards PDP**	Yes, it is necessary	2.10	<.001	[1.94, 2.27]
	No, not necessary*			

**Note**: * = Reference category.

## Discussion

To sustain the progression in public health and enhance children’s healthcare, while also working towards achieving Goal 16.2 of the Sustainable Development Goals (SDG)—which aims to eliminate all forms of violence against children in both public and private spheres—it is crucial to identify vulnerable groups and strengthen intervention programs by addressing the specific needs of each group in Bangladesh. This study found a significant relationship between physical disciplinary practices at home and factors such as the household’s financial status, the family’s residing region, type of residence, the child’s gender, and the mother’s education level and attitudes towards physical disciplinary practices. These findings suggest that targeted intervention efforts should be tailored to address the specific needs of these vulnerable groups.

In terms of household wealth or socioeconomic status, children from poor households are reported to experience significantly higher PDP compared to children from wealthier households. This disparity may stem from the economic stressors faced by lower socioeconomic status households, which heighten frustration and aggression among caregivers. Financial strain amplifies stress levels, shaping parenting behaviors and coping strategies, with PDP often perceived as a way to assert control or manage challenging situations. Such practices perpetuate a harmful cycle of violence within these households.

Conversely, economic stability can facilitate access to resources that support non-violent parenting, such as parenting classes and mental health services. Wealthier families may experience fewer resource-related conflicts and are better equipped to meet their own and their children’s needs, thereby reducing the frustration often associated with financial strain. This, in turn, may lead to less PDP as studies on children from Egypt support that household financial condition acts as a protection against PDP [52]. In Bangladesh, poverty is closely linked to the inability to meet basic needs, leading to frustration and stress for earning adults. Consequently, dependents such as children, the elderly, and disabled individuals may be viewed as burdens, resulting in PDP. Additionally, there is a prevailing belief in many areas that child-rearing requires physical discipline. Policies aiming to eliminate traditional norms in these areas, along with poverty reduction, should be considered to address these issues. Policies aimed at poverty alleviation, such as conditional cash transfers (CCTs), can be effective in breaking this cycle. For instance, CCT programs in countries like Brazil (Bolsa Família), Mexico (Oportunidades/Prospera), and Columbia (Familias en Acción) have successfully reduced child labor and improved educational outcomes, which indirectly reduced household stress and violence [53,54]. By providing financial support contingent on positive parenting behaviors, families can gain access to resources that reduce stress and promote healthier parenting practices. Additionally, implementing employment and skill development programs tailored to low-income caregivers can alleviate economic pressures and improve overall family dynamics.

The findings also collaborate with the results of a study in Egyptian children that male children are more vulnerable to physical violence at home than female children [52]. This pattern has been observed in various studies on corporal punishment of children globally [55,56]. For instance, research conducted in New Delhi schools found that boys were subjected to corporal punishment more frequently than girls, reflecting societal beliefs that boys require stricter discipline to develop assertiveness and dominance [57]. Similarly, a study in Pakistan also identified gender-based differences in corporal punishment [58]. In Bangladesh, this trend may be influenced by cultural perceptions that boys are more resilient and, therefore, can endure harsher disciplinary measures. This belief, coupled with societal expectations for boys to exhibit strength and independence, often leads to the justification of stricter punishment for their upbringing. Such practices reflect a broader societal tolerance for physical discipline as a means of instilling discipline and control.

The findings of the present study reveal a significant association between the type of residence and the use of physical disciplinary practices. Specifically, children residing in urban areas are more likely to experience PDP compared to those living in other settings. This trend may be attributed to factors such as economic pressure, housing density, and limited access to social services, which can exacerbate parental stress and lead to harsher disciplinary practices. Many studies have also highlighted the influence of a family’s place of residence on the types of disciplinary practices parents choose to use. For instance, research has shown that mothers in Palestine often resort to more frequent and severe forms of physical discipline, a trend attributed to the difficult conditions they face in their environment [59]. Similarly, another study in Uganda revealed that urban households faced greater stress due to socioeconomic disparities and overcrowded living conditions, which contributed to higher rates of corporal punishment compared to rural areas. Urban-focused interventions are essential to address these challenges. Programs that improve access to mental health services and parenting resources tailored to the unique stressors of urban environments, such as overcrowding, can help mitigate the use of PDP. Additionally, regional pilot programs targeting areas like Khulna and Sylhet, where the prevalence of PDP against children is highest, should combine economic support with parenting education to effectively reduce violence against children. Similar strategies have shown promise in high-stress urban communities in low-and middle-income countries, where tailored parenting programs reduced corporal punishment rates and improved parent-child relationships [60].

Previous research consistently indicates that violence against children is more common among mothers with lower levels of education [35,61,62]. The findings of the current study also indicate that physical disciplinary practices are associated with lower educational levels of mothers. This may be because mothers with little or no formal education are often unaware of the negative long-term effects of PDP against their children and may be more influenced by traditional norms that view PDP as necessary for child-rearing. This highlights the importance of maternal empowerment through education. Educated mothers are generally more aware of the detrimental effects of physical discipline on child development and are more likely to adopt positive parenting practices. Interventions that raise awareness among less-educated parents about the long-term consequences of physical discipline can significantly reduce violence against children. Such programs should focus on alternative, non-violent disciplinary methods and the benefits of positive reinforcement. By equipping mothers with knowledge and skills, we can promote the understanding that effective parenting does not depend on PDP. Additionally, increasing maternal education can boost women’s confidence and decision-making within the family, enabling them to advocate for healthier parenting practices. Creating supportive environments for mothers to share experiences and seek guidance can help break the intergenerational cycle of violence, ultimately improving children’s well-being in Bangladesh and fostering nurturing home environments.

Our results also align with the understanding that endorsing violence as a disciplinary method perpetuates further violence. Specifically, mothers or caregivers who believe that PDPs are necessary for effective child-rearing are more likely to use such methods, compared to those who do not hold this belief. Supporting this view, a study exploring the attitudes of Turkish parents toward physical disciplinary actions found that those who considered physical punishment necessary were more likely to practice it at home [63]. When caregivers believe that PDPs are necessary and see it as effective in some cases, it reinforces their belief system. If they perceive short-term compliance or behaviour change from their children, they might see this as validation of their approach, continuing the practice, and ignoring the long-term abuses. Also, the cultural normalization of PDP as a standard or acceptable method of discipline, makes it more challenging for caregivers to question or move away from such practices.

### Limitations

The mothers or caregivers answered the questions about physical disciplinary practices and not the children themselves; therefore, the prevalence of physical disciplinary practices could be under-reported. This could potentially lead to misleading study findings. To address this, future studies could incorporate complementary data collection methods, such as child interviews, or proxy reporting by other household members. Additionally, ensuring participant confidentiality and employing culturally sensitive phrasing in survey instruments may help minimize under-reporting. The short reference period of one month before the survey could also contribute to the under-reporting. The frequency of different forms of physical disciplinary practices was also overlooked due to a lack of available data in the measurement. Furthermore, the analysis was limited to identifying the determinants rather than the causes of the act due to the use of cross-sectional observational data.

## Conclusion

Bangladesh has made significant progress in reducing violence against children and women in the 21st century. However, additional efforts are required to meet the global standards set for achieving the Sustainable Development Goals (SDGs) [47]. This study aims to outline the profile of the most vulnerable groups at risk of experiencing PDPs by examining relevant sociodemographic factors and understanding how socioeconomic status influences the outcomes of such practices. Given that data on this issue may be underreported, the actual incidence could be higher, the findings indicate a pressing need for dedicated interventions and targeted policy frameworks to protect children from physical violence at home, with a particular focus on those from urban areas, especially those in poorer and underprivileged households.

Awareness and social education campaigns have proven effective in challenging traditional norms by redefining gender roles, empowering women, changing attitudes, promoting non-violent discipline, raising awareness of women’s and children’s rights, encouraging legal reforms, and fostering community support. Since physical disciplinary practices and poverty are closely linked, policies that target vulnerable groups at risk of physical violence and aim to improve children’s health are likely to enhance overall well-being. Such interventions could contribute significantly to achieving the United Nations’ Sustainable Development Goal 16.2, which focuses on ending abuse, exploitation, trafficking, and all forms of violence against children [47].

For future research, it would be advantageous if MICS continued to gather data on physical disciplinary practices against children. This would enable further studies to track trends in domestic physical disciplinary practices against children in Bangladesh and facilitate comparisons over time to support evidence-based policy-making. Additionally, there is a need for more in-depth qualitative research to explore why parents might initially prefer physical disciplinary methods. Investigating Bangladeshi mothers’ and caregivers’ perceptions of the effectiveness of various disciplinary practices, and whether they view violent practices as child maltreatment, could provide valuable insights into the high prevalence of physical discipline among Bangladeshi children.

## Acknowledgments

The authors gratefully acknowledge the Bangladesh Bureau of Statistics (BBS) and UNICEF for conducting a nationwide survey and providing open access to the data.
